# The Bronchiectasis Exacerbation Diary: a novel patient-reported outcome for non-cystic fibrosis bronchiectasis

**DOI:** 10.1183/23120541.00712-2022

**Published:** 2023-05-02

**Authors:** Vivian H. Shih, Maria Jison, Erik Bark, Meredith Venerus, Oren Meyers, James D. Chalmers

**Affiliations:** 1 Patient Centered Science, BioPharmaceuticals Medical, AstraZeneca, Gaithersburg, MD, USA; 2 Late Stage Respiratory and Immunology, BioPharmaceuticals R&D, AstraZeneca, Gaithersburg, MD, USA; 3 Patient Centered Science, BioPharmaceuticals Medical, AstraZeneca, Gothenburg, Sweden; 4 Patient Centered Solutions, IQVIA, Madrid, Spain; 5 Division of Molecular and Clinical Medicine, University of Dundee, Dundee, UK

## Abstract

Bronchiectasis is a chronic, progressive lung disease believed to result from a vicious cycle of infection and inflammation, with symptoms of chronic cough with sputum production, chronic fatigue, rhinosinusitis, chest pain, breathlessness and haemoptysis. There are currently no established instruments to monitor daily symptoms and exacerbations for use in clinical trials. Following a literature review and three expert clinician interviews, we conducted concept elicitation interviews with 20 patients with bronchiectasis to understand their personal disease experience. Findings from literature and clinician feedback were used to develop a draft version of the Bronchiectasis Exacerbation Diary (BED), which was designed to monitor key symptoms on a daily basis and during exacerbations. Patients were eligible to be interviewed if they were US residents aged ≥18 years, had a computed tomography scan–confirmed diagnosis of bronchiectasis with ≥two exacerbations in the previous 2 years and had no other uncontrolled respiratory conditions. Four waves of five patient interviews each were conducted. Patients (n=20) had a mean±_SD_ age of 53.9±12.8 years, and most were female (85%) and white (85%). A total of 33 symptoms and 23 impacts arose from the patient concept elicitation interviews. The BED was revised and finalised based upon patient feedback. The final BED is a novel, eight-item patient-reported outcome (PRO) instrument for monitoring key exacerbation symptoms on a daily basis with content validity established through comprehensive qualitative research and direct patient insight. The BED PRO development framework will be completed following psychometric evaluations of the data from a phase 3 bronchiectasis clinical trial.

## Abstract 2

**The Bronchiectasis Exacerbation Diary is a novel, eight-item, daily patient-reported outcome instrument for monitoring bronchiectasis exacerbation symptoms, with content validity established through comprehensive qualitative research with patient insight** https://bit.ly/3Hpwvn8

## Introduction

Bronchiectasis (or non-cystic fibrosis bronchiectasis) is a chronic, progressive lung disease believed to result from a vicious cycle of infection and inflammation, which leads to permanent bronchial dilation and bronchial wall thickening and impairs mucus clearance from the airways [1–3]. Ultimately, the disease may progress to respiratory failure and/or death. Patients with bronchiectasis typically present with primary symptoms of chronic cough with sputum production and frequent respiratory infections; secondary symptoms may include chronic fatigue, rhinosinusitis, chest pain, breathlessness and haemoptysis [2]. Although bronchiectasis is a heterogeneous condition with various aetiologies, including genetic abnormalities, immunological or autoimmune conditions, obstructing airway lesions, chronic aspiration and previous infections, the disease is considered idiopathic in up to 60% of patients [2, 4]. Even among patients who have the same underlying cause, the disease varies in severity and impact, and symptoms and exacerbations can be driven by different inflammatory profiles. Neutrophilic and eosinophilic phenotypes have been described [5, 6].

An international expert group recently developed a consensus definition for bronchiectasis exacerbations for use in clinical trials [7]. They defined a bronchiectasis exacerbation as a deterioration in at least three of six key symptoms (*i.e.*, cough, sputum volume/consistency, sputum purulence, breathlessness/exercise tolerance, fatigue/malaise and haemoptysis) for at least 48 h and a clinician determination that a change in bronchiectasis treatment is required [7]. There are no approved treatments for bronchiectasis, but several potential therapies are under evaluation in clinical trials with primary end points such as the frequency of exacerbations and time to first exacerbation [8–10]. Although several patient-reported outcome (PRO) measures are currently available to assess symptoms and quality of life in patients with bronchiectasis (*e.g.*, the Quality of Life–Bronchiectasis, the Bronchiectasis Health Questionnaire, the Bronchiectasis Impact Measure, and the Bronchiectasis Exacerbation and Symptom Tool), there are currently no content-valid instruments to monitor daily symptoms and exacerbations for use in clinical trials [11–14]. Current instruments used in the bronchiectasis population are not optimal for this purpose for several reasons, such as long recall periods, coverage of both symptoms and impacts (*i.e.*, health-related quality of life), and/or focus on a single symptom. Therefore, a fit-for-purpose PRO instrument is needed to specifically capture critical bronchiectasis symptoms on a daily basis to monitor patient health and detect worsening of daily symptoms, which may lead to an exacerbation [15]. The objective of this study was to understand patient perspectives on bronchiectasis symptoms and to use the collected data to develop a patient-centred, content-valid, US Food and Drug Administration (FDA)-compliant PRO instrument to monitor key symptoms on a daily basis and during exacerbations.

## Material and methods

### Patient eligibility and recruitment

Patients were eligible for the study if they were US residents aged ≥18 years, had a diagnosis of bronchiectasis (confirmed by computed tomography (CT) scan) with ≥2 exacerbations and/or hospitalisations in the previous 2 years and had no other uncontrolled respiratory conditions. Key inclusion and exclusion criteria are detailed in table 1, and full inclusion and exclusion criteria are included in the supplementary table. Best efforts were made to recruit patients with two-lobe involvement and a blood eosinophil count of ≥150 cells·μL^−1^ and to exclude patients with cardiac disease (*i.e.*, cor pulmonale, class III or IV congestive heart failure, symptomatic right ventricular failure, symptomatic uncontrolled cardiac arrythmias, pulmonary oedema in the past 4 weeks or cardiomyopathy), clinically important pulmonary disease other than bronchiectasis (*e.g.,* pulmonary fibrosis, cystic fibrosis, hypoventilation syndrome associated with obesity, lung cancer, α−1 anti-trypsin deficiency or primary ciliary dyskinesia), allergic bronchopulmonary aspergillosis requiring systemic steroid treatment in the past 6 months or radiological findings suggestive of another respiratory disease.

**TABLE 1:** Important inclusion and exclusion criteria

**Inclusion criteria**	**Exclusion criteria**
• Aged ≥18 years • Diagnosis of bronchiectasis o Confirmed by CT scan o ≥12 months since diagnosis o ≥2 bronchiectasis exacerbations or hospitalisations within the past 2 years • Able to participate in a 60–90-min interview to discuss signs, symptoms and impacts related to experience with bronchiectasis and to provide feedback on a draft PRO instrument • US resident	• Current or former smoker with a tobacco history of ≥10 pack-years • Current diagnosis of asthma and/or COPD that is NOT considered stable on maintenance treatment • Active tuberculosis • Active lung infection that has not been clinically resolved • Established clinical diagnosis of EGPA or HES • Long-term treatment with oxygen >4.0 L min^−1^

CT: computed tomography; PRO: patient-reported outcome; EGPA: eosinophilic granulomatosis with polyangiitis; HES: hypereosinophilic syndrome.

Patients were recruited by Global Perspectives using clinician referrals and social media outreach. Interested patients were informed about how to contact Global Perspectives, who then explained the study, confirmed the patient's willingness to participate *via* patient completion of an online or paper pre-consent form, and scheduled and conducted a screening call to determine patient eligibility. The interviews required patients to have access to a telephone and online screening platform.

### Ethics

Following the screening call, each patient who participated in the study was required to provide written informed consent; their clinician was required to provide written confirmation of their diagnosis. After patient eligibility was confirmed with the necessary documentation, Global Perspectives scheduled an interview for the patient. Patients received an honorarium for their participation.

Ethics approval was obtained from the New England Institutional Review Board (IRB number: 120190514). This study was conducted in accordance with the ethical principles that have their origin in the Declaration of Helsinki, consistent with Good Clinical Practice and applicable regulatory requirements, as well as in accordance with the regulations of the US FDA as described in 21 CFR 50 and 56, applicable laws and the IRB requirements.

### Study design and methods

**FIGURE 1:**
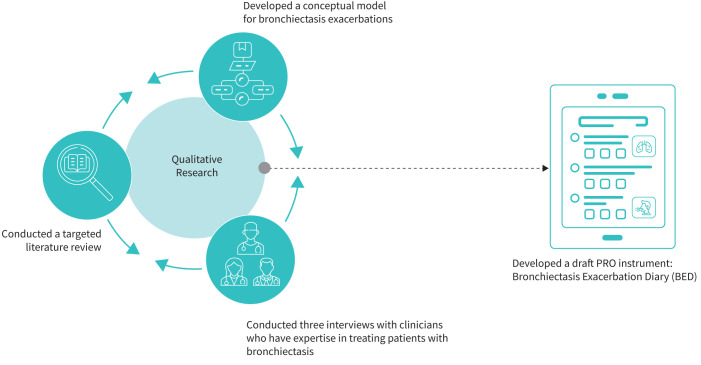
Development of the draft Bronchiectasis Exacerbation Diary. PRO: patient-reported outcome.

**FIGURE 2:**
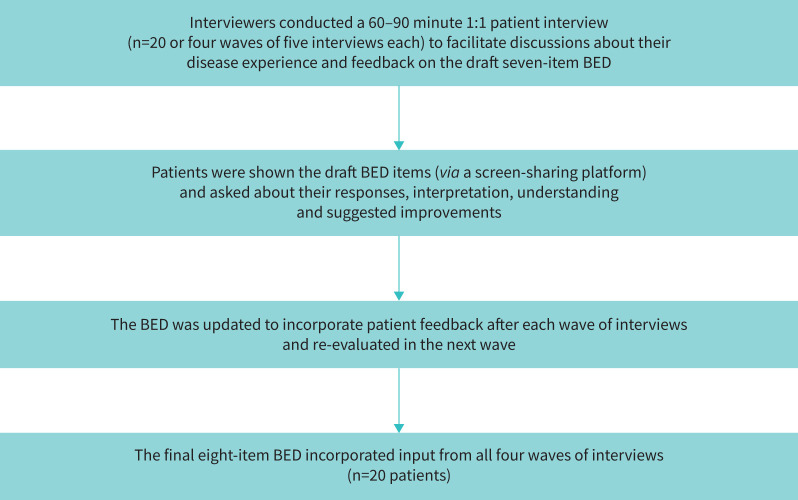
Workflow for patient interviews. BED: Bronchiectasis Exacerbation Diary.

The Bronchiectasis Exacerbation Diary (BED) PRO instrument was developed by AstraZeneca in collaboration with IQVIA through comprehensive qualitative research in accordance with FDA guidance, including a targeted literature review and three semi-structured interviews with clinicians in Europe who had expertise in respiratory conditions and experience of working with patients with bronchiectasis, with a goal of developing a preliminary bronchiectasis conceptual model (figure 1) [16]. The development of the preliminary conceptual model was an iterative process that incorporated key findings from the literature and clinician insight into the patient experience with bronchiectasis. Patient interviews (60–90 min long) were then conducted to revise and adjust the conceptual model based on the patient experience and to debrief the BED instrument (figure 2). Three (IQVIA) interviewers trained in interviewing patients for concept elicitation and cognitive interviews conducted the interviews.

The objectives of the patient interviews were as follows:

1. identify the symptoms of bronchiectasis experienced by patients and understand the impacts of bronchiectasis on overall patient health and functioning;
2. determine the words and phrases used by patients to describe their bronchiectasis experience;
3. document the patient experience of symptoms and impacts identified through interviews, especially those related to exacerbations;
4. update the conceptual model by incorporating direct patient feedback and finalise the BED; and
5. assess the content validity of the BED among patients with bronchiectasis and revise as appropriate.

A pre-specified, semi-structured patient interview discussion guide was provided to interviewers to ensure study objectives were met in a consistent manner. Additional details about the patient interview discussion guide are included in the supplementary material. It contained open-ended questions to avoid bias and support a free-flowing discussion between the interviewer and the patient. Patient interviews consisted of two sections: concept elicitation and cognitive debriefing. Concept elicitation interviews were aimed at characterisation of symptoms and impacts related to bronchiectasis, including timing and triggers, evolution over time, degree of disturbance (scale of 0 to 10, with 10 being the most disturbing) and effect on the patient's life. The cognitive debriefing interviews assessed the patient's comprehension of the BED instrument, including instructions, instrument items, response scales and recall period, and provided an opportunity for patients to make suggestions for improvement of the instrument.

### Data analysis

De-identified transcripts were coded using a qualitative coding dictionary, and concepts were categorised into symptoms and impacts. For each concept (*i.e.,* symptoms and impacts), counts of the number of patients who mentioned the concept (including total, spontaneous and probed mentions) and the mean disturbance ratings on a scale from 0 (not disturbing at all) to 10 (extremely disturbing) were tabulated. Additional information about coding is reported in the supplementary material. Concepts were considered salient if they were mentioned by ≥50% of patients and had a mean disturbance rating of ≥5 out of 10. Salient concepts were considered important components of the patient experience and were therefore prioritised for inclusion in the finalised BED. To assess evidence of concept saturation, the 20 interviews conducted were split into four chronological waves consisting of five patients each. Saturation was defined as the point at which conducting additional patient interviews did not introduce new concepts.

## Results

A total of 20 US patients with bronchiectasis were interviewed between January and July 2020. Patients were a mean±sd age of 53.9±12.8 years, and most were female (85%) and white (85%; table 2). Patients primarily had either one-lobe (45%) or two-lobe (40%) involvement, and 35% of patients had comorbid but medication-controlled asthma and/or COPD.

**TABLE 2:** Patient demographics and clinical characteristics (n=20)

**Category**	**Patients, n (%)**
**Age distribution**	
** **18–29 years	1 (5)
** **30–39 years	2 (10)
** **40–49 years	4 (25)
** **50–59 years	6 (30)
** **60–69 years	6 (30)
** **70–79 years	1 (5)
**Sex**	
** **Female	17 (85)
** **Male	3 (15)
**Ethnicity**	
** **White	17 (85)
** **Black or African American	2 (10)
** **Asian	1 (5)
**Highest level of education completed**	
** **High school	1 (5)
** **Some college/university	1 (5)
** **4-year college/university degree	9 (45)
** **Graduate degree or higher	6 (30)
**Lobe involvement**	
** **1 lobe	9 (45)
** **2 lobes	8 (40)
** **3 lobes	1 (5)
** **Bilateral lower lobe	2 (10)
**Key comorbidities**	
** **Controlled asthma	5 (25)
** **Controlled COPD	3 (15)
** **Arthritis	3 (15)
** **Coeliac disease	1 (5)
**>2 exacerbations and/or hospitalisations in the previous 2 years**	20 (100)
**>12 months since diagnosis**	20 (100)

**FIGURE 3:**
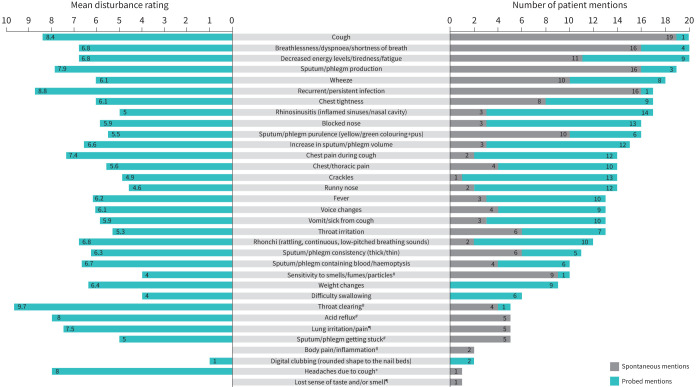
Mean disturbance rating (left) and number of patient mentions (right) for signs and symptoms of bronchiectasis. ^#^: concept was identified during wave 1 of patient interviews; ^¶^: concept was identified during wave 3 of patient interviews; ^+^: concept was identified during wave 4 of patient interviews.

Most symptoms (91%) of bronchiectasis were identified during the first-wave interviews (n=5 patients) (figure 3). Five new symptoms (*i.e.,* symptoms not identified during the targeted literature search or clinician interviews) were introduced during wave 1: throat clearing, sputum/phlegm getting stuck, acid reflux, body pain/inflammation and sensitivity to smells/fumes/particles; no new symptoms were mentioned during wave 2; and two new symptoms emerged during wave 3: lost sense of taste/smell and lung irritation/pain. During wave 4, headache due to cough was discussed as a new symptom by one patient; however, this concept was linked to the primary symptom of cough. Therefore, concept saturation of symptoms was achieved after wave 3. Similarly, most impacts (96%) of bronchiectasis were identified during the first wave of interviews (n=5 patients). Only one new impact was discussed in wave 2, and no new impacts emerged during waves 3 or 4. Therefore, concept saturation of impacts was achieved after wave 2.

A total of 33 symptoms arose from the concept elicitation interviews, with three symptoms reported by all patients: cough, breathlessness and fatigue (figure 3). A total of 23 symptoms were reported by at least 50% of patients, seven of which were spontaneously reported: cough, breathlessness, sputum/phlegm production, recurrent/persistent infection, fatigue, wheeze and sputum/phlegm purulence (figure 3, right). According to patient interviews, the symptoms most frequently experienced during a bronchiectasis exacerbation were cough (n=12), fever (n=12), and sputum/phlegm purulence (n=11) (table 3). Salient symptoms with the highest disturbance ratings were recurrent/persistent infection (mean rating of 8.8, rated by n=17 patients), cough (8.4, n=20), sputum/phlegm production (7.9, n=19), decreased energy levels/tiredness/fatigue (6.8, n=20), breathlessness/dyspnoea/shortness of breath (6.8, n=20), increase in sputum/phlegm volume (6.6, n=15), wheeze (6.1, n=18), chest tightness (6.1, n=17) and blocked nose (5.9, n=16) (figure 3, left).

**TABLE 3:** Top symptoms and impacts related to non-cystic fibrosis bronchiectasis exacerbations according to patient interviews (n=20)^#^

**Category**	**Patient mentions n (%)**
**Cough**	12 (60)
**Fever^¶^**	12 (60)
**Sputum/phlegm purulence (yellow/green colouring+pus)**	11 (55)
**Decreased energy levels/tiredness/fatigue**	8 (40)
**Sputum/phlegm consistency (thick, thin)**	8 (40)
**Wheeze**	7 (35)
**Rhonchi**	6 (30)
**Sputum/phlegm production**	6 (30)
**Breathlessness/dyspnoea/shortness of breath**	4 (20)
**Sputum/phlegm containing blood/haemoptysis**	4 (20)
**Chest/thoracic pain**	3 (15)
**Crackles**	3 (15)
**Increase in sputum/phlegm volume**	3 (15)
**Runny nose**	3 (15)

Note: rows highlighted in green were included in the final eight-item BED. BED: Bronchiectasis Exacerbation Diary. ^#^: blocked nose, chest pain/tightness, throat and lung irritation, rhinosinusitis, voice changes and vomiting were also associated with exacerbations by one to two patients. ^¶^: although “fever” was also expressed by patients to be indicative of an exacerbation, it was determined that a more objective method of measuring patient temperature would be more accurate and appropriate.

A total of 23 impacts arose from the concept elicitation interviews, with the most frequently experienced impacts (*i.e.*, experienced by 90% of patients) being sleep disturbance/poor sleep quality and breathlessness during or after activities/low exercise tolerance (supplementary figure, right). 20 impacts were reported by at least 50% of patients, seven of which were spontaneously reported: breathlessness during activities/low exercise tolerance, embarrassment due to cough and sputum/phlegm production, avoidance of activities, decreased ability to work or do housework, hospitalisations, frustration due to condition and modification/limitation of activities/holidays/social activities. The impacts with the highest mean disturbance ratings were hospitalisations (8.6, n=10), embarrassment due to cough and sputum/phlegm production (8.1, n=14), frustration due to condition/cough (7.6, n=15), daytime sleepiness (7.5, n=12), impact on relationships (7.3, n=15), fear of infection (6.9, n=14) and financial impacts (6.9, n=14) (supplementary figure, left).

**FIGURE 4:**
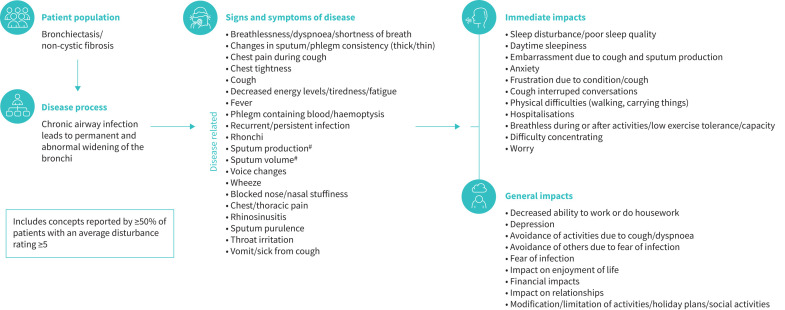
Final bronchiectasis conceptual model: salient concepts. ^#^: sputum production refers to the act of coughing up sputum, whereas sputum volume refers to the amount of sputum.

The bronchiectasis conceptual model was finalised after incorporating feedback from the patient interviews (figure 4).

Patients also provided feedback on the BED instrument during the cognitive debriefing interviews. Most patients (95%) felt that a 24-hour recall period was appropriate for answering the items in the BED instrument. In waves 1 and 2, patients (n=10) evaluated the original draft of the BED instrument, and most reported that they understood what each item was asking and were able to provide a response. However, half of patients (n=5) in waves 1 and 2 gave feedback regarding ways to improve the clarity of the BED. A definition of mucus (phlegm) was added to the BED to help anchor patients prior to answering questions related to their experience with mucus. A new gateway question was also added to assess if the patient had experienced mucus in the past 24 h; if they had not, they would skip the items assessing mucus amount, colour and thickness and move on to the next item on haemoptysis. Because of the addition of the gateway questions, the option of “no mucus” was removed from all mucus-related items in the BED. To account for patients who do not regularly look at their mucus, “I have not noticed the colour of my mucus” was added as a response to the mucus colour item. Instructions were added at the beginning of the BED to help patients understand the purpose of answering the questions in BED and to explain that the questions target symptoms specifically related to their bronchiectasis.

**FIGURE 5:**
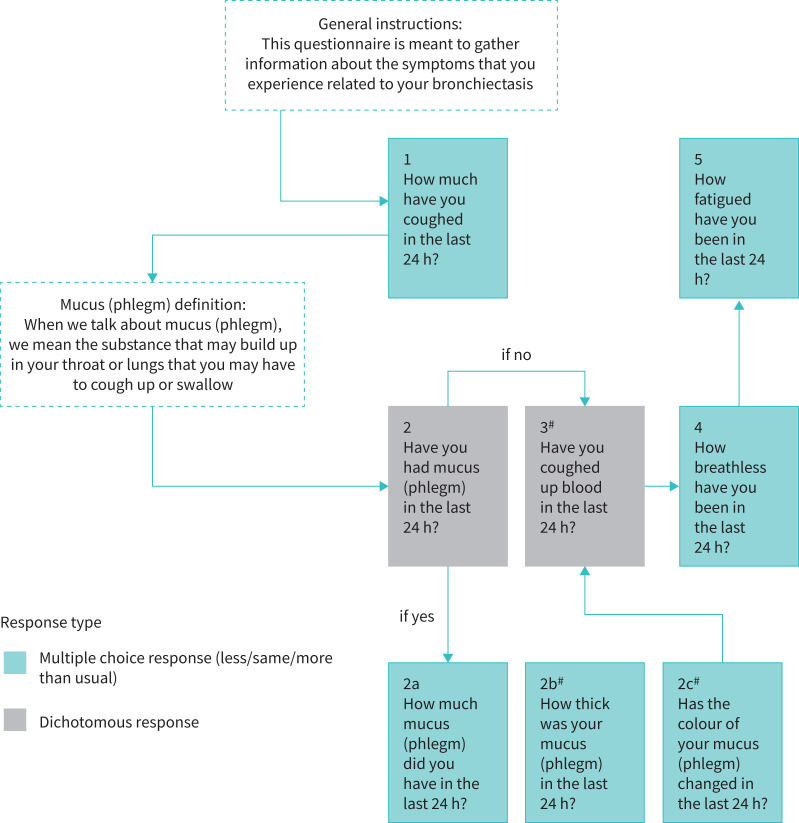
Final daily eight-item Bronchiectasis Exacerbation Diary. ^#^: includes “have not noticed” as a response option.

In wave 3, patients (n=5) reviewed a revised draft of the BED instrument incorporating feedback from the previous waves; most patients reported that the BED instrument was easy to understand and the new items were well defined. Following wave 3, “I have not noticed” was added as a response to two additional items related to coughing up blood and the thickness of mucus, and these changes were incorporated and re-evaluated in wave 4. Overall, patients (n=5) in wave 4 understood the items and instructions. The only change following wave 4 was the removal of the term “non-cystic fibrosis” from the instructions. In all, the final eight-item BED instrument was updated to include additional instructions, one gateway question and alternative response options based on iterative waves of patient interviews and direct patient feedback (figure 5).

## Discussion

Health authorities and professional societies emphasise the value of measuring and accounting for the patient experience during the drug development process. PRO instruments are developed with the input of existing literature, clinicians, patients and psychometric experts to describe and standardise the patient experience of various aspects of a disease or its treatment, such as disease symptoms, adverse drug effects or functional outcomes. PRO instruments are increasingly being deployed in clinical trials as primary and secondary end points to enhance clinician understanding of disease or disease treatment aspects that are subjective or not directly observable [17].

20 patients with bronchiectasis were interviewed over four waves for the purposes of concept elicitation and cognitive debriefing related to development of the BED instrument. During the concept elicitation interviews, 33 symptoms were identified, 20 of which were salient, with concept saturation achieved after wave 3. Six key symptoms associated with a bronchiectasis exacerbation (*i.e.* coughing, sputum/phlegm production, sputum/phlegm purulence, breathlessness, fatigue and haemoptysis) were highlighted based on patient feedback, and these symptoms aligned with and further corroborated the expert consensus definition [7]. The BED instrument was revised iteratively and finalised after incorporating direct patient feedback. The comprehensive patient-centric qualitative findings support use of the content-valid BED instrument to capture bronchiectasis symptoms and support exacerbation end points in clinical studies. The BED is currently in use to capture symptom worsening and enable physicians to identify a potential bronchiectasis exacerbation, which may require a change in treatment, in the MAHALE study. MAHALE is a phase 3 study (NCT05006573) underway to evaluate the efficacy and safety of benralizumab in adults with bronchiectasis with eosinophilic inflammation.

It is essential for the development of new treatments to demonstrate accurately treatment effects on exacerbations and quality of life. A key finding of multiple bronchiectasis clinical trials has been a reduction in the exacerbation rate from the period before the study to the period during the study. Underreporting of exacerbations and a failure to identify symptom worsening accurately have been cited as possible reasons for this phenomenon, which leads to underpowering of clinical trials. The BED was designed to address this issue by providing a systematic capturing of symptoms and exacerbation-related symptom deteriorations on a daily basis. Experience in other conditions such as COPD suggests that such tools can identify unreported exacerbation events.

Patients interviewed for this study had similar characteristics to adult patients enrolled in bronchiectasis registries around the world, suggesting that this instrument could be deployed globally. In the US Bronchiectasis Research Registry (BRR), the European Multicentre Bronchiectasis Audit and Research Collaboration (EMBARC) and the Australian Bronchiectasis Registry (ABR), patients with bronchiectasis were predominantly female and white, and the most common comorbidities were asthma and COPD [3, 18–20]. A UK population-based study showed similar findings [21].

Our study had several limitations. The data collected did not include aetiology of bronchiectasis. Although the patients in our study with asthma and/or COPD were on stable maintenance treatments and the percentages of patients with these comorbid conditions were comparable to those in the BRR and ABR, patients could have experienced symptoms related to these comorbid respiratory condition(s) that they attributed to their bronchiectasis, or vice versa, or there may have been overlapping symptoms. Patients included in our study were generally younger than might be expected for a group with bronchiectasis, with 65% of patients under age 60 years, while the mean patient age was 64 years in the BRR and 65 years in EMBARC and the median patient age was 71 years in the ABR. More work will be needed to explore the use of the BED instrument in a broader and/or older patient population [3, 18, 20]. One potential explanation for the difference in patient age may be that some patient recruitment was performed during the initial lockdowns of the COVID-19 pandemic, a time during which older patients were visiting their clinicians less frequently. Additionally, one method of recruitment was *via* social media, and patients were required to have a telephone and internet connection for the interview, so these factors may have contributed to the slightly younger sample. Our interviewed patients were very highly educated, with 75% of patients having a bachelor's degree or higher, and although bronchiectasis is more common among patients of higher socioeconomic status, the high level of education could have influenced patients’ perceptions of the terminology used in the BED [21]. Finally, this study only included patients recruited from the USA. As such, the results may not be representative of other geographical areas, and caution should be applied when extrapolating the findings to other regions.

In this study, we performed the initial development and validation of the BED. However, we enrolled relatively few male patients, and our method of enrolment is likely to have skewed our patient population towards higher socioeconomic groups. In addition, bronchiectasis is a heterogeneous condition with diverse clinical, aetiological and microbiological characteristics that cannot be fully represented in a study of this kind. Because of these limitations and considerations, the BED now requires extended validation among diverse bronchiectasis populations on a large scale.

### Conclusions

The final BED is a novel eight-item PRO instrument for monitoring symptoms of a bronchiectasis exacerbation, which supports the component of the consensus exacerbation definition on symptom deterioration and fills an unmet need for a daily PRO symptom instrument. The content validity of the BED was established through comprehensive qualitative research, which included a literature review, clinician interviews and, most importantly, direct patient insight. This rigorous, stepwise process confirmed patient understanding and established the appropriateness of the BED for capturing the key symptoms associated with bronchiectasis exacerbations. The BED PRO development framework will be completed following psychometric evaluations of the data from the MAHALE study.

## Supplementary material

10.1183/23120541.00712-2022.Supp1 **Please note:** supplementary material is not edited by the Editorial Office, and is uploaded as it has been supplied by the author.
Supplementary material 00712-2022.SUPPLEMENT
