# Supplementary material for: The Bronchiectasis Exacerbation Diary: a novel patient-reported outcome for non-cystic fibrosis bronchiectasis
Source: ERJ Open Res. 2023 May 2;9(3):00712-2022. doi: 10.1183/23120541.00712-2022 (PMC10152244; doi:10.1183/23120541.00712-2022)

The Bronchiectasis Exacerbation Diary: A Novel PRO for Non-Cystic Fibrosis Bronchiectasis

Vivian H. Shih,[a] Maria Jison,[b] Erik Bark,[c] Meredith Venerus,[d] Oren Meyers,[d] James D. Chalmers[e]

[a]Patient Centered Science, BioPharmaceuticals Medical, AstraZeneca, Gaithersburg, MD, USA

[b]Late Stage Respiratory and Immunology, BioPharmaceuticals R&D, AstraZeneca, Gaithersburg, MD, USA

[c]Patient Centered Science, BioPharmaceuticals Medical, AstraZeneca, Gothenburg, Sweden

[d]Patient Centered Solutions, IQVIA, Madrid, Spain

[e]Division of Molecular and Clinical Medicine, University of Dundee, Dundee, UK

Corresponding author:

Vivian H. Shih, DrPH

Patient Centered Science, BioPharmaceuticals Medical, AstraZeneca

1 MedImmune Way

Gaithersburg, MD 20878 USA

E-mail: vivian.shih@astrazeneca.com

MATERIAL AND METHODS

Study design and methods

Patients were first asked open-ended questions about symptoms. For example, “What symptoms do/did you experience that you think are related to NCFB?” Patients were then asked to rate each spontaneously-reported symptom on a scale of 0 to 10 to indicate how bothersome each symptom was. Patients were also specifically asked about timing, duration, and frequency of each symptom. If symptoms on the interviewer guide were not spontaneously mentioned, patients were asked follow-up questions to probe for these symptoms and to rate any symptoms they confirmed they had experienced.

De-identified transcripts were generated from interview audio-recordings. ATLAS.ti v8 software was used to code all transcripts. The primary goals of transcript coding were to track the number of patients who mentioned a concept, track if the concept was first mentioned spontaneously or probed, organize and catalogue concept descriptions / quotes, and track disturbance ratings. Transcript coding was based on coding rules that were established before coding began and iterated throughout the coding process to ensure accuracy and consistency. The process of coding was distributed evenly between two coders to ensure Inter-coder Agreement (ICA) ≥0.7 was achieved. After the ICA threshold was achieved, approximately every fifth transcript was double coded to ensure consistency of coding. Concepts were categorized into two broad categories: signs/symptoms and impacts. For all concepts, counts of the number of patients who mentioned the concept (including total, spontaneous, and probed mentions), average disturbance ratings, and saturation analyses were computed.

Supplementary Table. Full inclusion and exclusion criteria

Inclusion criteria	Exclusion criteria
• Adults ≥18 years old<br>• NCFB diagnosis<br>o Confirmed by CT scan<br>o Have been diagnosed with NCFB for at least 12 months<br>o Have history of 2 or more NCFB exacerbations or hospitalizations within the past 2 years<br>• Patient is willing and able to provide informed consent to research<br>• Patient is legally and mentally capable of providing informed consent to research<br>• Patient is willing and able to participate in a single 60-90-minute interview to discuss signs, symptoms, and impacts related to their experience with NCFB and provide feedback on a draft PRO instrument<br>• Patient resides in any state in the US	• Patient has a mental disability or significant mental illness, legal incapacity or limited legal capacity or any other lack of fitness, which, in the opinion of the screener, would preclude the patient's participation in or ability to complete the study<br>• Current smokers or ex-smoker with a tobacco history of ≥10 pack-years history<br>• Current diagnosis of asthma, COPD or asthma-COPD overlap that is NOT considered stable on maintenance treatment<br>• Active tuberculosis<br>• Receiving long-term treatment with oxygen >4.0 litres/minute (L/min)<br>• Active lung infection that has not been clinically resolved<br>• An established clinician diagnosis of eosinophilic granulomatosis with polyangiitis or hypereosinophilic syndrome
On a best-effort basis, study recruitment will aim to recruit patients who also have:<br>• 2 lobe involvement<br>• Blood eosinophil count ≥ 150/µL	On a best-effort basis, study recruitment will aim to exclude patients who have:<br>• Cardiac diseases that include: cor pulmonale, congestive heart failure class III or IV, symptomatic right ventricular failure, symptomatic uncontrolled cardiac arrythmias, pulmonary oedema in the past 4 weeks, cardiomyopathy<br>• Allergic bronchopulmonary aspergillosis (ABPA) requiring systemic steroid treatment in the past 6 months<br>• Clinically important pulmonary disease other than bronchiectasis, including pulmonary fibrosis, cystic fibrosis, hypoventilation syndrome associated with obesity, lung cancer, alpha-1 anti-trypsin deficiency, and primary ciliary dyskinesia<br>• Radiological findings suggestive of a respiratory disease other than bronchiectasis

Supplementary Figure. Average disturbance rating (left) and number of patient mentions (right) for impacts of bronchiectasis

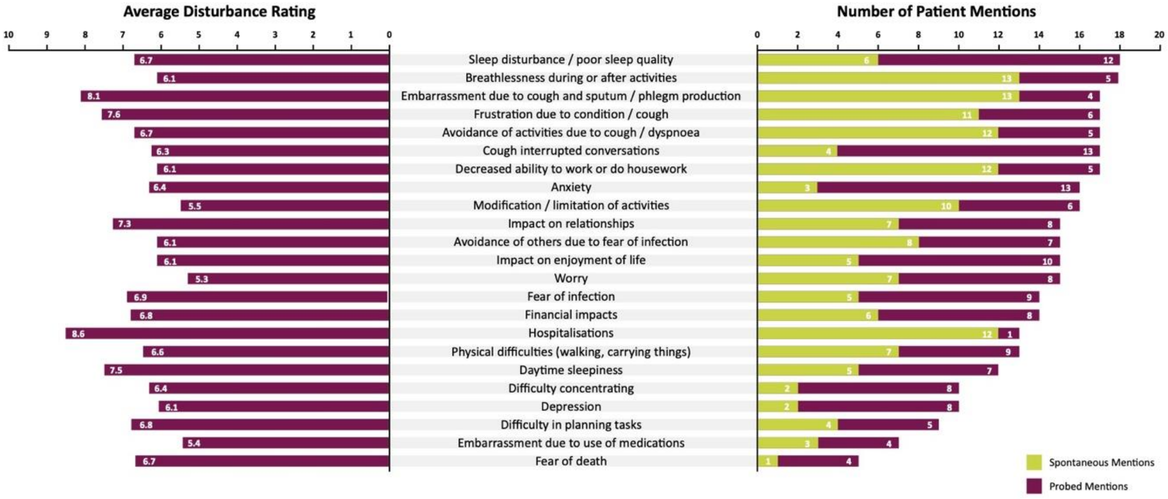

Supplement: Supplementary file 1 [file 00712-2022.SUPPLEMENT.pdf]
